# Supplementary material for: Covalent DNA Binding Is Essential for Gram-Negative Antibacterial Activity of Broad Spectrum Pyrrolobenzodiazepines
Source: Antibiotics (Basel). 2022 Dec 7;11(12):1770. doi: 10.3390/antibiotics11121770 (PMC9774941; doi:10.3390/antibiotics11121770)
Supplement: Supplementary file 1 [file antibiotics-11-01770-s001.zip › antibiotics-2038891-supplementary.pdf]

antibiotics

Supplementary File

Purity determination of synthesized final compounds

The level of purity of the compounds for biological testing has been evaluated through LC-MS analysis, using two different gradient methods, reported hereafter. LC-MS analyses were performed on a Waters Alliance 2695 system (from Waters), with elution in gradient. HPLC grade solvents were used as mobile phase while a Monolithic C18 50 X 4.60 mm column (from Phenomenex) was used as stationary phase. UV detection was performed using a Waters 2996 photo array detector (from Waters). Injection volume has been set to 10 μL. The compounds have been dissolved in a mixture of H_2O/ACN (50/50, v/v) or DMSO/ACN (50/50, v/v) accordingly to the solubility. The area of the peak corresponding to the compound has been automatically determined by the software included in the LC-MS system. The eventual presence of solvent UV trace has been subtracted to the total in order to determine the percentage of purity.

LC-MS methods:

Method A: flow 0.5 mL/min

A) water + 0.1 % formic acid

B) acetonitrile + 0.1% formic acid

**Time (min)**	**0**	**3**	**3.5**	**4.5**	**5**
**A (%)**	95	10	5	5	95
**B (%)**	5	90	95	95	5

Method B: flow 1 mL/min

A) water + 0.1 % formic acid

B) acetonitrile + 0.1% formic acid

**Time (min)**	**0**	**2**	**5**	**6**	**7.5**	**9**	**10**
**A (%)**	95	95	50	50	5	95	95
**B (%)**	5	5	50	50	95	5	5

**Compound**	**5 minutes method**		**10 minutes method**	
	**Retention time (min.)**	**Purity**	**Retention time (min.)**	**Purity**
**7a**	3.12	≥98%	5.95	≥98%
**8a**	3.30	≥98%	6.40	≥98%

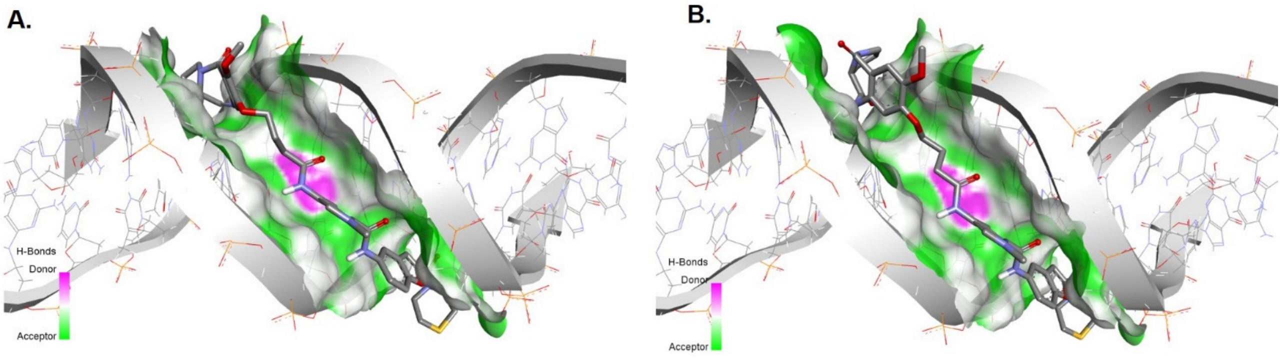

Figure S1. DNA binding of compounds 7 (A) and 7a (B) within the DNA minor groove of Sequence-2.

Compound 7

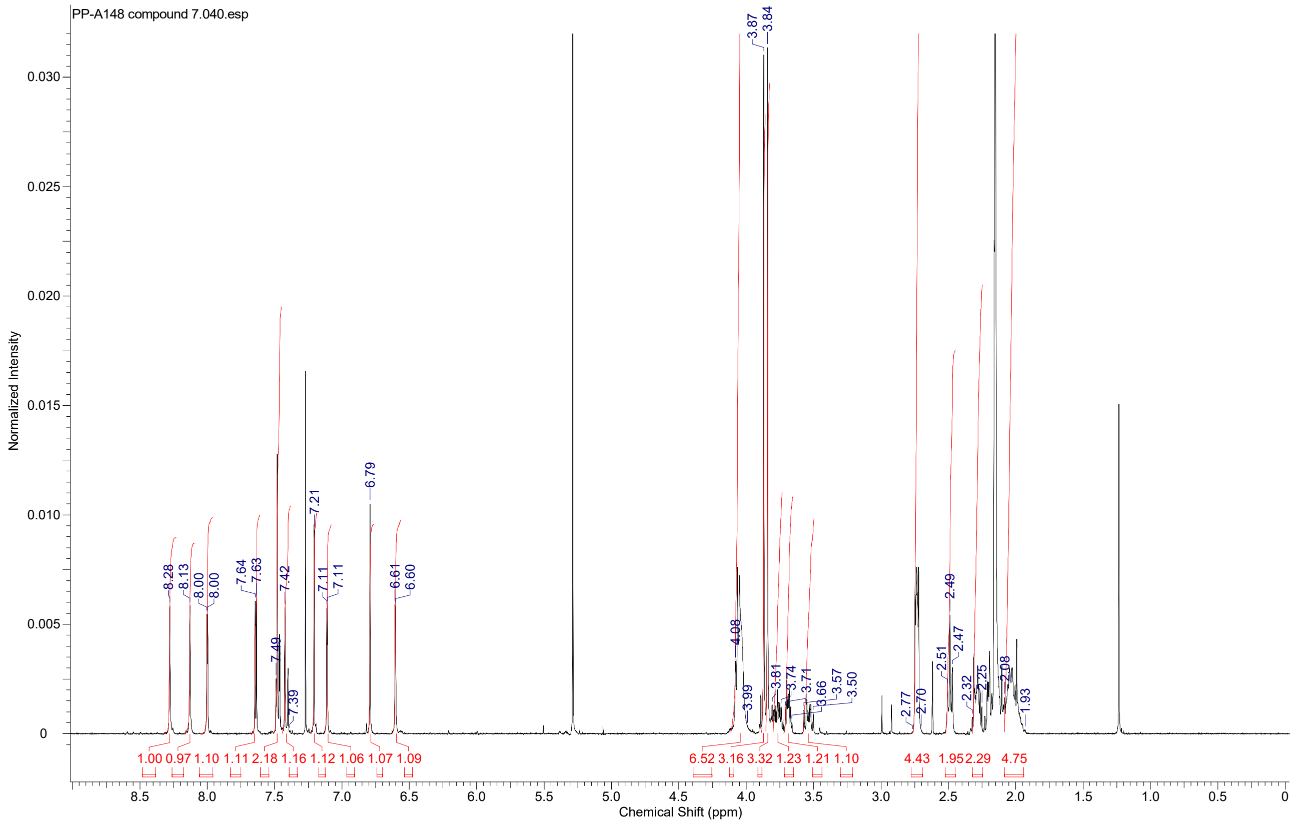

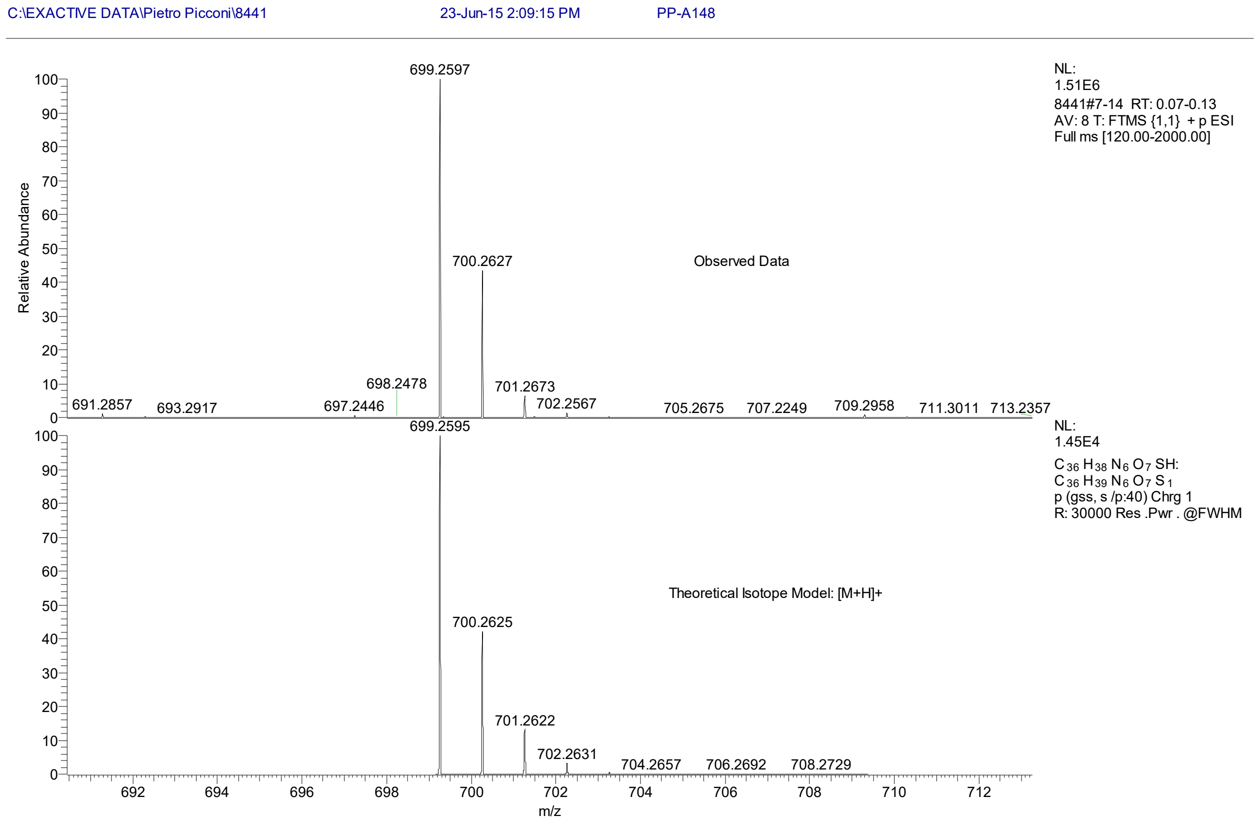

Figure S2. ^{1}HNMR and HRMS of compound 7.

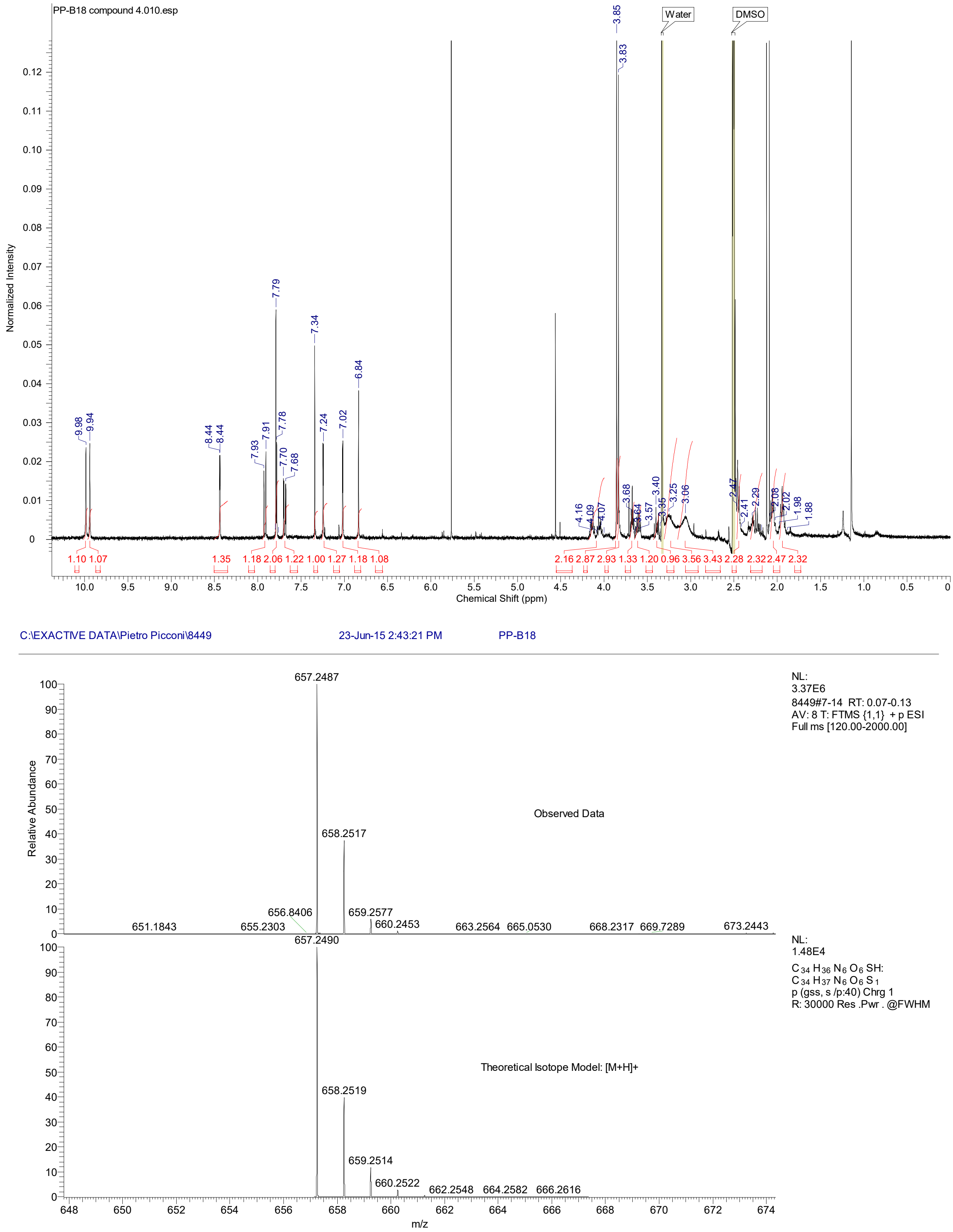

Figure S3. ^{1}HNMR and HRMS of compound 8.

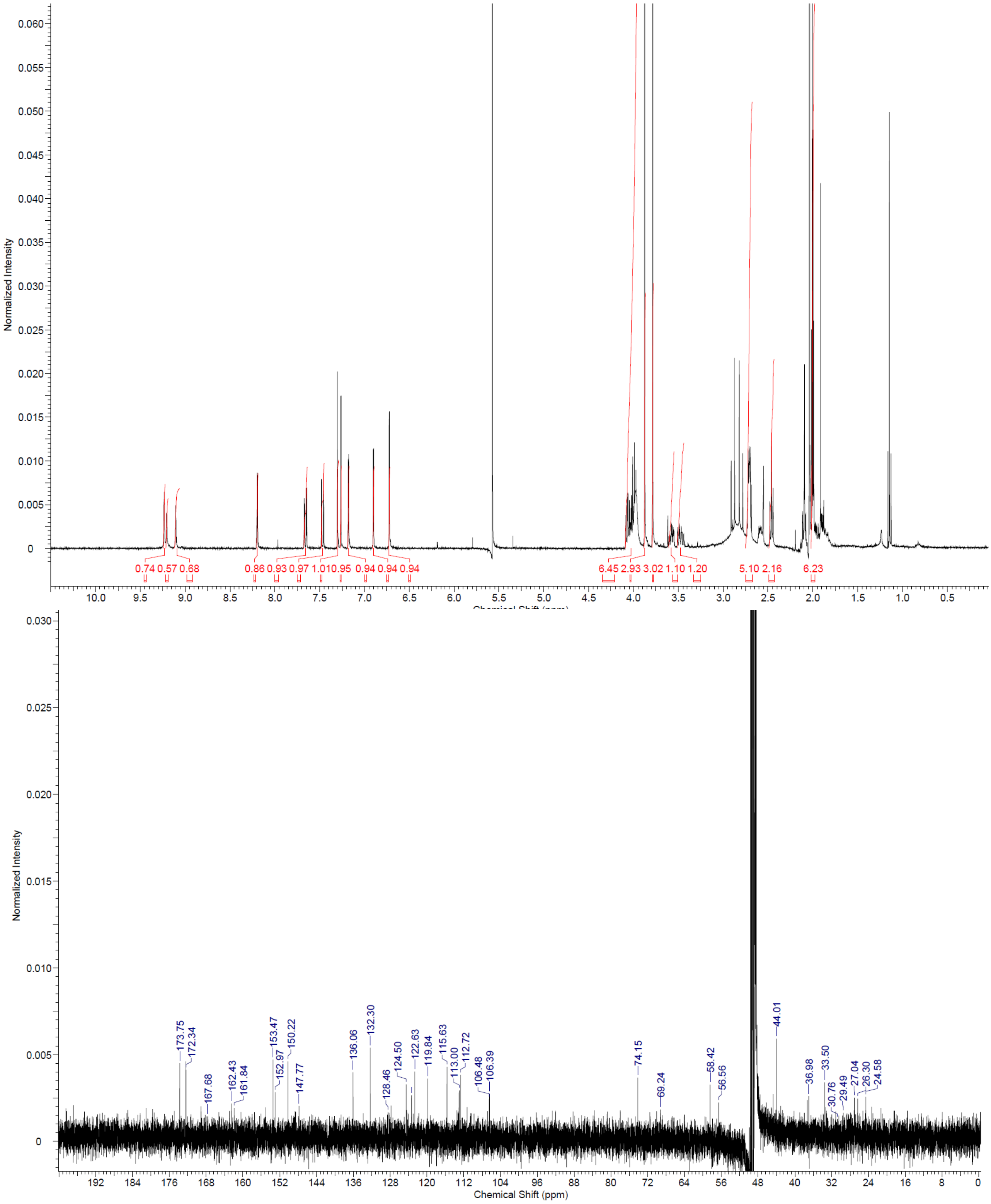

Figure S4. ^{1}HNMR and ^{13}CNMR of compound 7a.

Openlynx Report - Pietro Page 1
Sample: 1 Vial:1:F,5 ID:dilactam
File:Pietro3448-1 Date:26-Feb-2016 Time:12:31:05
Description:

Printed: Fri Feb 26 12:44:27 2016

Sample Report:

Sample 1 Vial 1:F,5 ID dilactam File Pietro3448-1 Date 26-Feb-2016 Time 12:31:05 Description

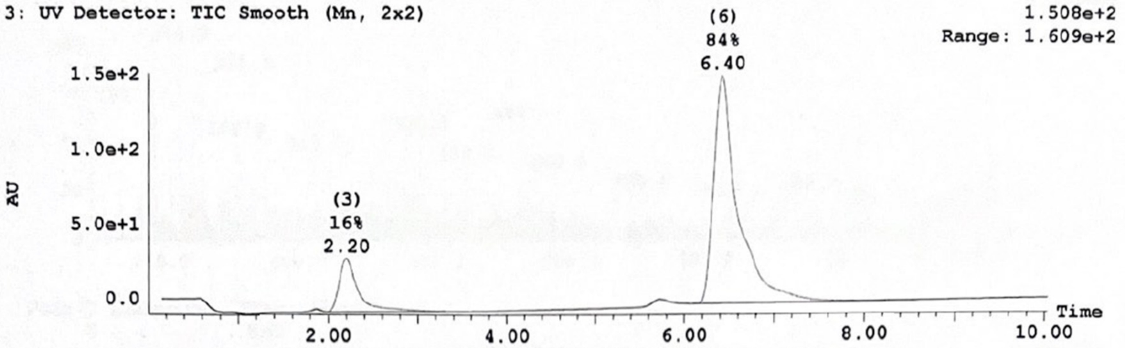

Peak Number	Compound	Time	AreaAbs	Area %Total	Width	Height	Mass Found
3		2.20	9e+006	15.95	1	4e+007	
6		6.40	5e+007	84.05	2	2e+008	

Openlynx Report - Pietro Page 2
Sample: 1 Vial:1:F,5 ID:dilactam
File:Pietro3448-1 Date:26-Feb-2016 Time:12:31:05
Description:

Printed: Fri Feb 26 12:44:27 2016

Sample Report (continued):

Peak ID	Compound	Time	Mass Found
3		2.20	

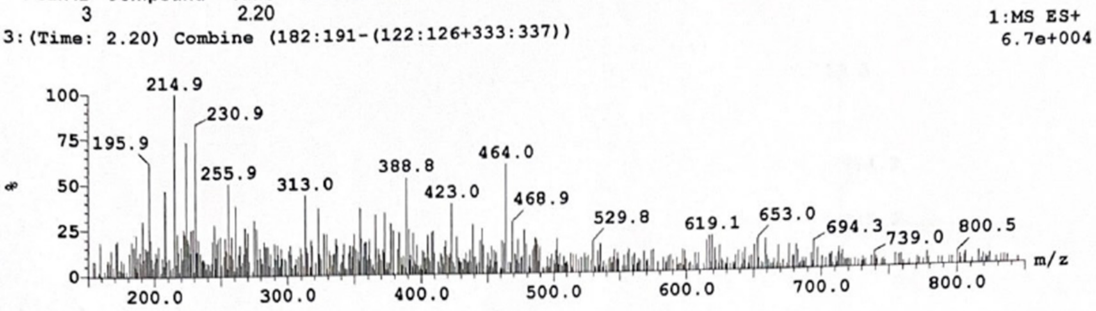

Peak ID	Compound	Time	Mass Found
6		6.45	

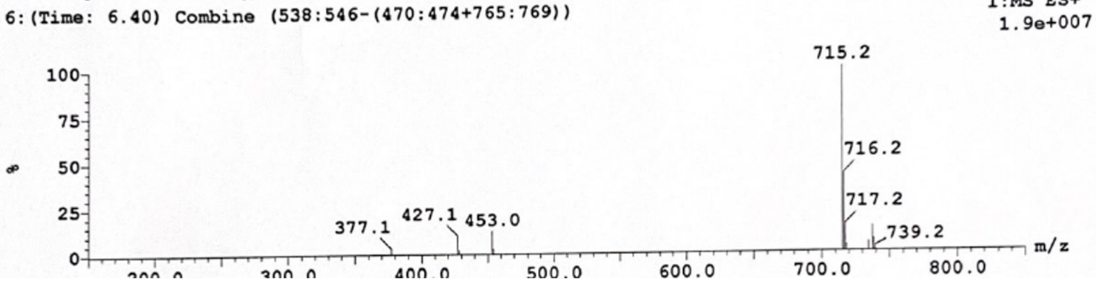

Figure S5. LCMS Profile of compound 7a (peak at RT 2.20 is the solvent front peak).

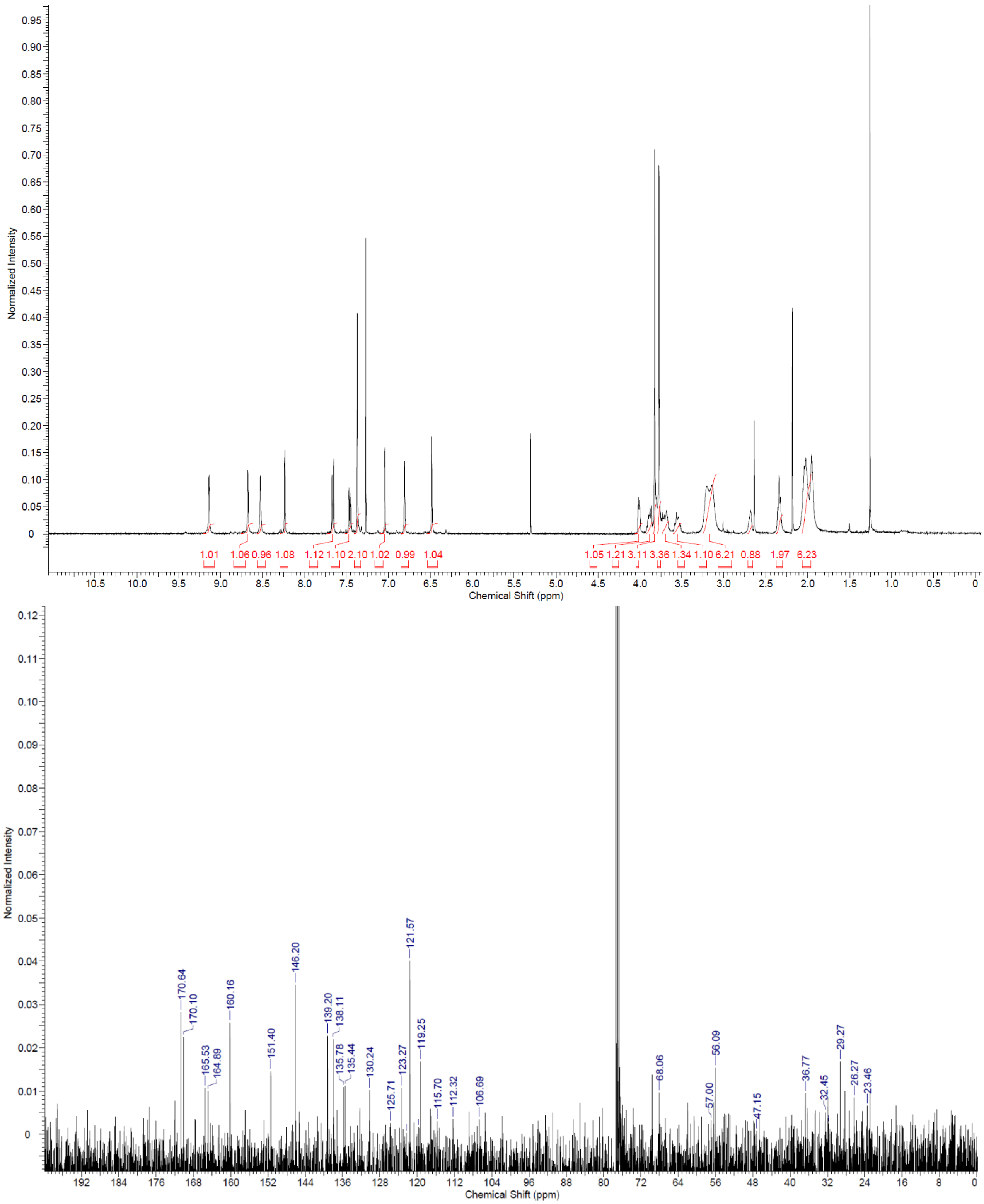

Figure S6. ^{1}HNMR and ^{13}CNMR of compound 8a.

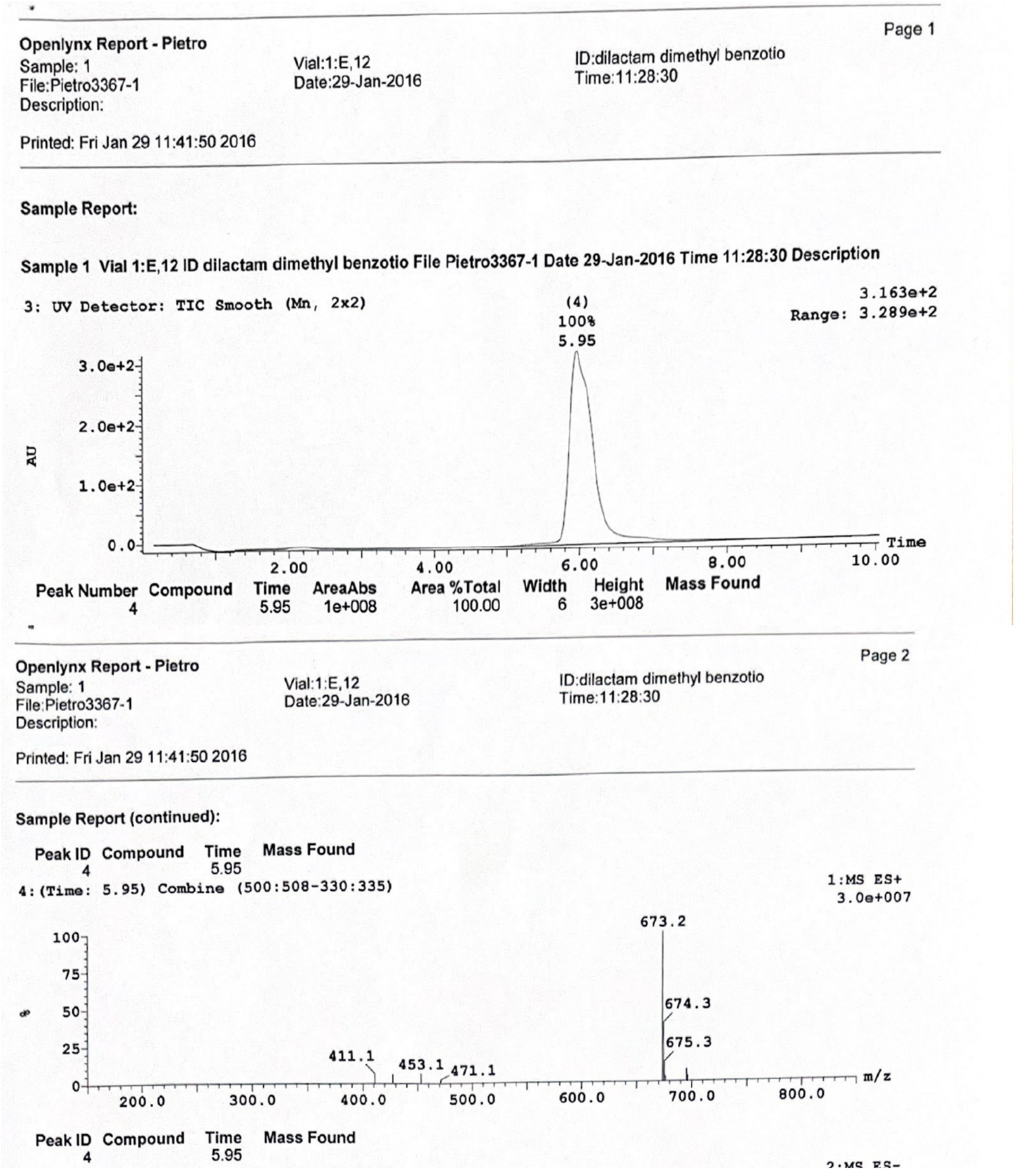

Figure S7. LCMS Profile of compound 8a.
